# Platelets as a Gauge of Liver Disease Kinetics?

**DOI:** 10.3390/ijms231911460

**Published:** 2022-09-28

**Authors:** Sheng-Hung Chen, Shih-Chang Tsai, Hsiu-Chen Lu

**Affiliations:** 1 Department of Medicine, China Medical University, No. 91, Xueshi Road, Taichung 404333, Taiwan; 2 Center for Digestive Medicine, Department of Internal Medicine, China Medical University Hospital, No. 2, Yude Road, Taichung 404327, Taiwan; 3 Department of Biological Science and Technology, China Medical University, Taichung 404333, Taiwan; 4 Department of Education, China Medical University Hospital, Taichung 404327, Taiwan

**Keywords:** platelet count, liver cirrhosis, monitoring, risk assessment, thrombopoietin, platelet aggregation, flow cytometry, elastography

## Abstract

A multitude of laboratory and clinical interferences influence the utility of platelet-based diagnostic indices, including immature platelet fraction, in longitudinal monitoring and prognostication of patients with chronic liver disease (CLD). The complex yet highly regulated molecular basis of platelet production and clearance kinetics becomes dysregulated in liver pathogenesis. These underlying molecular mechanisms, including premature platelet clearance and bone marrow suppression in parallel with the progressive (e.g., treatment-naïve) or regressive (e.g., on-treatment and off-treatment) disease courses, involved in CLDs, may further confound the changes in platelet–liver correlations over time. Platelet count and function are commonly and secondarily altered in vivo in CLDs. However, the precise characterization of platelet functions during cirrhosis, including in vitro platelet aggregation, has proven challenging due to interferences such as thrombocytopenia. A flow cytometric approach may help monitor the unstably rebalanced hyper- and hypoaggregable states in patients with cirrhosis at risk of hyperaggregable, prothrombotic, or bleeding events. Studies have attempted to stratify patients with cirrhosis by substages and prognosis through the use of novel indices such as the ratio of in vitro endogenous platelet aggregation to platelet count. This review attempts to highlight clinical and laboratory precautions in the context of platelet-assisted CLD monitoring.

## 1. Introduction

Resting thrombocytes or platelets are small (2–4 μm) discoid [1], anucleate cytoplasmic pieces that are shed by mature progenitor cells: megakaryocytes in the bone marrow after birth. Each megakaryocyte can release protoplatelets that break into 5000–10,000 platelets per megakaryocyte. Typically, an adult human produces approximately 100 billion platelets a day, and the production can increase 10–20-fold in emergencies via the initiation of megakaryocyte rupture by interleukin (IL)-1α [2]. Platelet production can further increase 5–10-fold via thrombopoietin (TPO)–myeloproliferative leukemia (Mpl) receptor axis signaling by exogenous TPO mimetic drugs or TPO receptor (TPO-R) agonists [3].

The life span of platelets is typically 7–10 days. The reference platelet count is widely quoted as being 150–400 × 10^9^/L of the whole blood by international guidelines and the latest research. Thrombocytopenia, defined as a platelet count below 150 × 10^9^/L, is the most common hematological abnormality encountered in patients with chronic liver disease (CLD) [4]. In patients with liver cirrhosis, thrombocytopenia develops due to decreased platelet production, increased splenic sequestration, and increased breakdown. For clinical monitoring, platelet count-based diagnostic indices [5], including the FIB-4 index, exhibit kinetics (typically declines in the FIB-4 index) in parallel with antiviral treatment courses in chronic viral hepatitis. From the pretreatment baseline, platelet count–based diagnostic indices exhibit valid performances in the diagnosis of concurrent liver fibrosis and in the prognosis of liver-related events (LREs), including portal hypertension–related events and hepatocellular carcinoma (HCC). However, platelet count measurements and platelet-based diagnostic indices encounter numerous laboratory pitfalls and clinical interferences that potentially impact their utility in patient monitoring [6]. The complex yet highly regulated molecular basis of platelet production and clearance kinetics become dysregulated with liver disease progression. These underlying molecular mechanisms, along with progressive (e.g., treatment-naïve) or regressive (e.g., on-treatment or off-treatment) courses, of liver disease confound the utility of and changes in platelet–liver correlations over time [7].

Endogenous circulating platelets are generally maintained in a quiescent phenotype by endogenous nitric oxide and prostacyclin released by intact vascular endothelium and by the downstream cyclic nucleotide-dependent pathways in platelets [8]. Conversely, upon blood vessel or tissue injury, platelets aggregate as the trigger of main platelet function or primary hemostasis. Platelet characteristics, including platelet shape, platelet granule content, and platelet adhesion receptor density, are fundamental for stable clot formation under high-flow conditions [9]. Beyond physiological aggregation, platelets also play crucial roles in thrombosis or secondary hemostasis [10,11], vascular tone and integrity, plasma homeostasis, tissue regeneration, ischemia–reperfusion injury, fibrosis, innate and adaptive immune responses, antimicrobial host defense, sepsis [12], disseminated intravascular coagulation (DIC), and metabolic disorder-related platelet hyperactivity. In the context of liver diseases, platelets, which serve as patrolling, immediate responders, interact intimately with resident parenchymal and nonparenchymal cells and nonresident cells of the liver, immune cells, and nonimmune cells including endothelial cells, pathogens, and tumor cells. Platelets counterbalance pathophysiological processes across various stages of liver fibrogenesis and in acute-on-chronic liver failure, nonalcoholic steatohepatitis, alcoholic liver disease, viral hepatitis, autoimmune hepatitis, liver carcinogenesis, regeneration, and immunity against cancer and metastasis. Platelets may exert both beneficial and harmful effects. As acute liver diseases [13] and CLDs progress or resolve, platelets are not only closely affected over time but also actively modulate critical hepatic events in the intravascular and extravascular milieu directly and indirectly, independent of their hemostatic function. These hepatic events include necroinflammation, fibrogenesis, steatosis, (lymph)angiogenesis, and immune regulation [14].

Besides primary platelet diseases, platelet count and function are commonly and secondarily altered in liver diseases. Liver fibrosis severity is critical to treatment decision-making and prognostication. Liver fibrosis stages correlate positively with the risk of treatment adverse events and LREs and negatively with treatment outcomes. At the stage of cirrhosis, in vitro platelet aggregation is debatable because the interpretation of platelet function is challenged by thrombocytopenia [15]. Moreover, aggregable, and thrombotic states may be unstably rebalanced to potentially hyperaggregable and prothrombotic cirrhosis [16,17]. Platelet function/metabolism-dedicated assays [18,19,20,21], including aggregometry, electron microscopy, flow cytometry, mass spectrometry, and “omics” studies, have been used in clinical and experimental settings to facilitate platelet-assisted evaluation of CLD kinetics, which may not be fully interpreted by concurrent platelet counting alone. However, the point-of-care utility of platelet-specific markers should be evaluated by prospective and retrospective studies on diagnostics, prognosis, and treatment monitoring in CLD over time. Such assays have been challenging to implement in patient cohorts worldwide, with archived cryopreserved blood samples alone [22].

**Figure 1:**
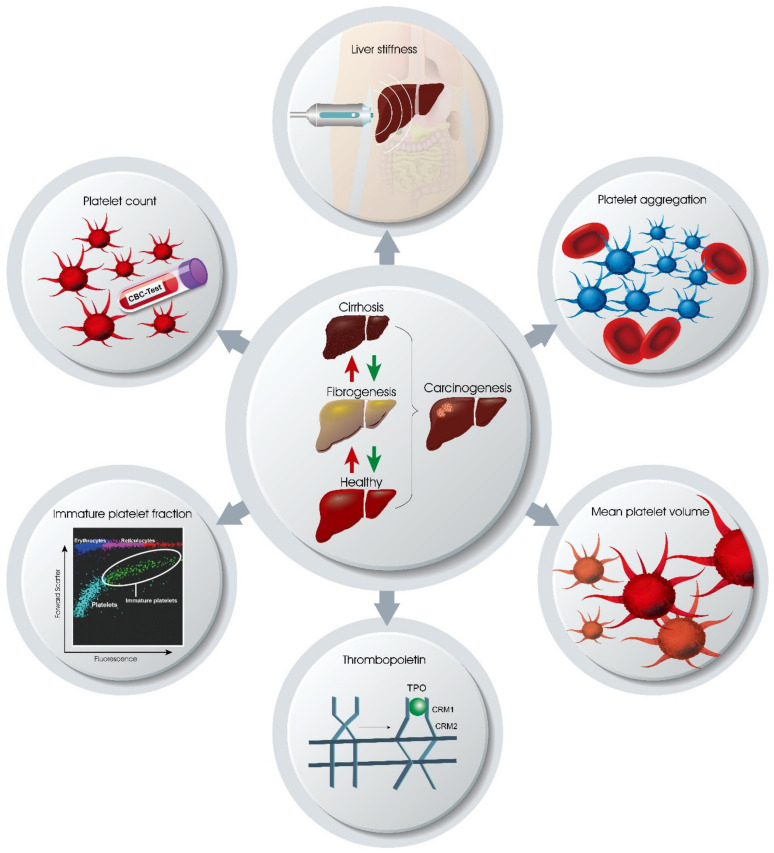
Platelet-based liver disease monitoring. Red arrows indicate disease progression. Green arrows indicate disease regression.

Therefore, this review provides updated information on in vitro candidate tests (Figure 1) for platelet-assisted CLD monitoring from samples to big data [23]. While elucidating the in vivo platelet–liver kinetics, the review results aimed to highlight clinically and experimentally available and applicable approaches in terms of the molecular basis, settings, disease indications, typical technical pitfalls and precautions, advantages and disadvantages, and clinical confounding factors in the context of CLD monitoring.

## 2. Thrombocytopenia in CLDs

### 2.1. Decreased Platelet Production

The glycoprotein cytokine, TPO, cloned in 1994, is a ligand that targets the c-Mpl proto-oncogene-encoded protein receptor TPO-R, which was cloned in 1990. TPO shares a significant amino acid sequence homology with erythropoietin. TPO is primarily produced in the liver, bone marrow, and kidneys by both parenchymal cells and liver sinusoidal endothelial cells (LSECs) and is secreted constitutively into the circulation. The 635-amino-acid-long TPO-R (CD110) has three functional domains: an extracellular portion involved in cytokine binding, a transmembrane domain, and a cytoplasmic domain that binds to JAKs and other signaling molecules, such as STATs. TPO-R is expressed on the surfaces of stem cells, megakaryocyte progenitor cells, megakaryocytes, and platelets. TPO regulates the development and maturation of megakaryocytes and the subsequent release of platelets [4]. When TPO binds to TPO-R, TPO-R is dimerized and the JAK family of nonreceptor tyrosine kinases, as well as the STAT family, the MAPK family, the adaptor protein Shc, and the receptors themselves become tyrosine phosphorylated. The activation of TPO-R on megakaryocytes activates hematopoiesis via the nonreceptor JAK2, tyrosine kinase 2 and downstream signaling pathways [24]. Generally, decreased liver reserves during the profibrotic disease process and (viral) hepatitis flares lead to decreased production of TPO and, consequently, platelets [25].

Bone marrow progenitor cells and their niche components are adversely and multifactorially affected in advanced chronic and acute liver diseases. Platelet production is decreased due to decreased hepatic TPO production and suppressed platelet production in the bone marrow, secondary to various pathogenesis, including viral infection, excess alcohol consumption, iron overload, and medications. A reported pathophysiology proposed that hepatitis B and C viruses may inhibit the growth and differentiation of human bone marrow progenitor cells, resulting in direct bone marrow hypoplasia. Hepatitis B and C viruses can directly infect the bone marrow, thereby inhibiting platelet production and accelerating premature platelet destruction by activating the monocyte–macrophage system and immune system [26,27]. Chronic heavy alcohol consumption can also lead to impaired bone marrow niches, increased mitochondrial dysfunction of bone marrow progenitors, ineffective megakaryopoiesis, and apoptosis of circulating platelets, independently of the secondary pathogenesis in relation to chronic alcoholic liver damage [28]. The kinetics of the effects of extrahepatic pathogenesis on platelets confound the utility of platelet counts in CLD monitoring.

### 2.2. Platelet Sequestration

Splenomegaly is an important feature of portal hypertension. Hypersplenism results in the genuine splenic destruction of platelets rather than mere splenic pooling or sequestration of platelets.

Vascular endothelial growth factor is a key factor that maintains the differentiated fenestrated phenotype of LSECs through endothelial nitric oxide synthase-derived nitric oxide signaling. Intrahepatic dysregulation, including the dedifferentiated capillarized phenotype of LSECs and the profibrogenic, proangiogenic phenotype of hepatic stellate cells, contributes to increased intrahepatic resistance. Portal hypertension is the consequence of pathologically increased intrahepatic resistance, extrahepatic splanchnic hypocontractibility and hyperperfusion, and pathologically altered intrahepatic and extrahepatic vasculature [29].

Intrahepatic microvascular thrombosis can aggravate the progression of portal hypertension and liver fibrosis in CLDs and acute liver failure. Rivaroxaban, an anticoagulant drug that inhibits an active form of factor X, thereby inhibiting thrombin formation, attenuated liver fibrosis and portal hypertension in cirrhotic rats (induced by thioacetamide and CCl4), likely by reducing intrahepatic endothelial dysfunction, HSC activation, and microvascular thrombosis rather than via a direct antifibrotic effect [30]. However, clinical evaluation of effects of rivaroxaban use on platelet counting is unclear.

### 2.3. Platelet Destruction

In CLD, increased platelet destruction may be due to cirrhosis-related hypersplenism with sequestration and destruction of platelets, immune-mediated platelet destruction such as with hepatitis C virus infection, and intrahepatic and extrahepatic intravascular platelet consumption [31] as occurs in DIC [32].

Physiologically, as platelets become apoptotic or senescent in blood vessels, or after accomplishing their main function in the bloodstream, platelets can be removed from the circulation by neutrophils or macrophages, including resident Kupffer cells, and destroyed or phagocytosed in the spleen or liver. Platelet apoptosis depends on the balance between the proapoptotic and antiapoptotic signaling pathways in platelets. Antiapoptotic Bcl-2 family proteins, including Bcl-2, Bcl-w, and Bcl-xL, block the proapoptotic molecules Bak and Bax, which can initiate mitochondrial damage. BH3-only proteins, including Bid, Bim, Bad, and Bik, which are inhibitors of the antiapoptotic Bcl-2 family proteins, can, together with mitochondrial permeabilization, decrease the mitochondrial electrochemical gradient and release of cytochrome C, redistribution of phosphatidylserine from the inner to the outer leaflet of the platelet plasma membrane, and phosphatidylserine exposure, contributing to apoptotic platelet clearance [7]. Desialylation of O-glycans on platelet glycoprotein (GP)Ibα drives receptor signaling and platelet clearance. The binding of soluble plasma activators, including von Willebrand factor (vWF) or antibodies, to the ligand-binding domain of GPIbα on platelets can activate GPIb-IX under high shear stress by triggering unfolding of the mechanosensory domain of the GPIb-IX complex, thereby inducing downstream signaling in the platelets, including desialylation. Deglycosylated or hyposialylated platelets are recognized by one of the C-type lectins, the Ashwell-Morell asialoglycoprotein receptor (AMR), and potentially other scavenger receptors on hepatocytes and macrophages and are rapidly cleared by them [33]. In contrast, platelet clearance inhibitors, such as neuraminidases and GPIbα shedding inhibitors, maintain platelet viability both in vivo and during storage [20].

## 3. Are Platelets a Promising Gauge of CLD Kinetics?

### 3.1. Liver Stiffness Measurement

#### 3.1.1. Molecular Basis

Patient preference for invasive liver tissue biopsy has been decreasing. Liver stiffness is a well-known, promising biomarker that can be measured through diverse noninvasive modalities that exhibit comparable diagnostic performances. Liver stiffness is a combination measurement of real-time liver status that must be addressed to serve as a reference test for the evaluation of “platelet–liver kinetics” during CLD progression or regression. Liver stiffness is primarily modulated by interrelated components, including the intrahepatic extracellular matrix’s collagen content; the dynamic component of hepatic sinusoidal pressure comprising blood flow, intrahepatic resistance, and hemorheology; and the static component of pressure related to vasculature elasticity and intravascular volume filling status. Liver stiffness also results from intrahepatic intracellular pressure through transport proteins and aquaporins as well as stretch forces on the cellular membranes and intermediary filaments of the parenchymal hepatocytes and nonparenchymal hepatic stellate cells and LSECs. When liver stiffness occurs, the mechanosensing of hepatic parenchymal and nonparenchymal cells drives various mechanosignaling pathways. Potential mechanosensing mechanisms include cation channels of the transient receptor potential family, the actin-interacting protein zyxin, and G-protein-coupled receptors activated in response to stretching. In contrast, ion channel activation and alterations in cytoskeletal stability are part of the response to hydrostatic pressure [34]. As platelet counts decline during CLD progression, concurrent liver stiffness is upregulated. Conversely, liver stiffness progression or regression mainly reflects the ongoing stage of liver fibrosis and the degree of portal hypertension, which attenuate or restore platelet counts over time [35].

#### 3.1.2. Liver Stiffness Kinetics in CLD

Real-time liver stiffness can therefore be used to assess the changes in liver status; however, the measurement technology is pending certain improvement in a minority of clinical and research conditions exhibiting excessive measurement variabilities and compromised measurement validity. Liver stiffness can commonly be affected by clinical features, including concurrent hepatic necroinflammation, intrahepatic and extrahepatic cholestasis, hepatic congestion, active alcohol consumption, immediate postprandial state, and relevant medications in an examinee. The measurements may be complicated by variations in measurement results (typically, increases in values) that compromise measurement validity (Table 1). A thorough clinical context should be incorporated into decision algorithms.

In general, liver stiffness rapidly declines with the early, rapid resolution of hepatic necroinflammation following the initiation of treatment for chronic hepatitis B (CHB) and chronic hepatitis C (CHC); by contrast, the subsequent slow phase of decreasing liver stiffness reflects the sustained but slow regression of liver fibrosis. Theoretically, posttreatment, noninvasive cutoff values of liver stiffness deduced for evaluating posttreatment, concurrent, and “left-over” morphometric liver fibrosis stages in invasive liver histology exhibiting sparse necroinflammation should be lower than those cutoffs at baseline in treatment-naïve patients.

Posttreatment liver-related adverse events, including hepatic carcinogenesis and portal hypertension-related complications, have been reported worldwide among patients achieving viral eradication. These complications can be primarily related to residual posttreatment liver fibrosis, which may contribute to pivotal upstream molecular mechanisms (Table 2). With the variations in liver stiffness values measured over time from the viremic to nonviremic states, posttreatment liver stiffness values may concurrently reflect the genuine burden of real-time remnant liver fibrosis. By mitigating the confounding effects of hepatic necroinflammation on liver stiffness measurements over time, posttreatment liver stiffness values are thus promising to classify posttreatment liver fibrosis stages and cirrhosis substages and to identify patients at high risk of liver-related adverse events. The baseline values of liver stiffness for a posttreatment surveillance program should be acquired after treatment. Absolute liver stiffness values acquired off treatment may compensate for the missed early surveillance on-treatment, thereby allowing for long-term surveillance. Various studies have indicated that [36,37] a posttreatment liver stiffness value above a threshold during surveillance serves as a red flag to alert both physicians and patients that activation is mandatory for a prespecified recall policy comprising intense screens and surveillance during lifelong risk stratification.

### 3.2. Platelet Counting

#### 3.2.1. Preanalytical Errors

To follow up on changes in liver disease severity over time through platelet-based diagnostic indices (references) on and off liver disease treatments, accurate and reliable peripheral blood platelet count is imperative to avoid erroneous risk stratification and over- or undertreatment for a liver disease. Before validations, preanalytical, sampling, and technical errors must be ruled out through a prespecified algorithm based on technical or biological decision trees or machine learning modeling. Ensemble modeling that incorporates features in terms of sampling and technical details, past health-care history, clinical context, concurrent surrogate biomarkers, and liver pathology may assist in identifying the over- or underestimations of platelet counts and proposing a recall policy in advance of spurious reporting, including pseudothrombocytopenia, while differentiating between productive and consumptive etiologies of thrombocytopenia (Table 1).

The best alternative anticoagulant in the case of typical EDTA (EDTA)-induced pseudothrombocytopenia remains unclear. An EDTA tube requires 8–10 inversions to mix blood thoroughly with the anticoagulant. Erroneous thrombocytopenia may be reported due to improper blood sampling, preanalytical storage, filling, and difficult or inadequate inversions of the test tubes [54]. If a coagulated sample indicating preanalytical noncompliance is detected in a test tube, the counting must be rejected. If preanalytical abnormalities are associated with the reference anticoagulant EDTA, a second sample run can be performed with another anticoagulant in addition to a new EDTA tube count tested after adequate inversions at 37 °C in parallel. If platelet satellitism or platelet clumping is observed on the peripheral blood smear, the sample could be recollected using alternative anticoagulants, including citrate, heparin, and oxalate. Platelets can then be counted using an automated method. Platelet satellitism or granulocyte-platelet rosettes are a rare in vitro acquired phenomenon, typically associated with EDTA-treated blood at room temperature. Granulocyte-platelet satellitism may involve the platelet membrane GPIIb/IIIa complex and IgG autoantibodies against a cryptic antigen on the platelet GPIIb/IIIa complex and on the neutrophil Fc gamma III receptor (CD16). These antibodies may induce adhesions between platelets and lymphocytes, lymphoma cells, and even bacteria. In addition, EDTA chelates calcium ions while causing the dissociation of the platelet membrane GPIIb/IIIa complex. The consequent decrease in calcium concentration reveals the hidden epitope of GPIIb. EDTA-dependent antiplatelet autoantibodies can bind to EDTA-modified epitopes, thereby triggering platelet aggregation. Antiplatelet antibodies can also trigger platelet activation via tyrosine kinase, leading to platelet clumps and, therefore, to the reporting of false thrombocytopenia in vitro. Pseudothrombocytopenia due to EDTA-induced clumping, large platelets, or platelet cold agglutinins can be confirmed by examining a stained peripheral blood smear [38].

#### 3.2.2. Technical Incapacities

In addition to the aforementioned sampling-related concerns, currently available hematology analyzers have employed impedance, optical methods including light scattering or fluorescence techniques, and immunofluorescence techniques using monoclonal antibodies directed against the membrane GPs of platelets. Which alternative technique should be preferred in the case of analytical interferences? One example of interference is that impedance-based analyzers enumerate the platelet numbers by size. However, interference may come from the sizes. A false increase in platelet count may occur in the presence of nonplatelet particles the size of platelets, such as fragmented erythrocytes, cytoplasmic fragments of nucleated cells, bacteria, fungi, cryoglobulins, and lipids. Conversely, a false decrease in platelet count can be encountered with giant or large platelets, rosettes, and clumps. In the case of persistent interference, immunological platelet counting may ultimately serve as the reference method while calibrating the automated hematology analyzers in patients with cirrhosis suspected of thrombocytopenia. However, this immunological method requires a flow cytometer, which needs to be operated by experienced technicians. Platelets are labeled by fluorescein-conjugated monoclonal antibodies against two distinct epitopes of the platelet integrin αIIbβ3 (CD41 and CD61) and analyzed using a flow cytometer to calculate the fluorescence-emitting platelets to RBC ratio in the same suspension [38].

Delta check is a process used to detect discrepancies in patient test results prior to reporting by comparing current patient values to previous ones. A smear review is mandatory for a significant change in platelet counts without obvious cause (delta > 50% compared with a recent preceding result in adults). However, in the case of severe thrombocytopenia (<20 × 10^9^/L), such as in cirrhosis, the delta check would be meaningless. A method for monitoring platelet clearance is to measure the lifespan of endogenous platelets. A radioisotopic or fluorescent compound is administered to pulse-label the circulating platelets. Platelets are isolated from the whole blood sampled periodically. The percentage or radioactivity of labeled platelets in the whole platelet population in vitro is measured and plotted over time. Moreover, Plateletworks, a point-of-care rapid in vitro platelet function test that performs whole-blood hematology analysis, can measure changes in platelet counts before and after platelet aggregation through platelet stimulation by agonists, including ADP, thrombin receptor-activating peptide, arachidonic acid, collagen, and collagen-related peptide. Platelet aggregation is evaluated by calculating the percent maximal platelet aggregation using a minimal volume of citrated whole blood. Plateletworks is primarily used for surgery and cardiology to monitor antiplatelet therapies. However, it is an indirect assay that requires accurate platelet counting [55]. Few studies have explored its utility in platelet-assisted CLD monitoring.

#### 3.2.3. Confounding Clinical Context

##### Factors Increasing Platelet Counts

Numerous clinical features may also confound decision algorithms. For example, certain drugs may1 alter platelet counts through thrombocytogenic effects. Drug-induced thrombocytosis is a rare but possible complication. The most robust evidence of causality supports low-molecular-weight heparin, aside from heparin-induced thrombocytopenia (HIT). Heparin can also induce thrombocytosis by potentiating megakaryopoiesis—in particular, by inhibiting platelet factor 4 (PF4). Thrombocytosis is another reason to monitor platelet counts during heparin treatment. Weaker evidence exists for all-transretinoic acid, antibiotics, clozapine, epinephrine, gemcitabine, and vinca alkaloids. From viremic to nonviremic status through antiviral treatments in patients with CHB or CHC, the molecular mechanisms underlying the prompt restoration in platelet production within 1 month after the start of treatments is warranted. The AMR can bind and remove desialylated platelets and regulate hepatic TPO production by activating JAK2/STAT3, thereby regulating platelet production. After partial hepatectomy, platelet counts were rapidly restored by upregulation and crosstalk of the AMR and the IL-6R to induce JAK2–STAT3–TPO activation in the liver, accompanied by an increased number of megakaryocytes in the spleen and bone marrow before the liver was completely regenerated [39]. Moreover, platelets are acute-phase reactants; therefore, reactive or secondary thrombocytosis may develop in response to various stimuli, including systemic infections, inflammatory conditions, bleeding, and tumors. Multiple types of cancer cells and metastatic dissemination may release thrombopoietin and other growth factors that induce the maturation and mobilization of megakaryocytes from the bone marrow, leading to increased circulating platelet mass as a paraneoplastic syndrome [56].

##### Factors Decreasing Platelet Counts

Conversely, in patients with untreated CHB or CHC and cirrhosis, a decrease in platelet counts can be detected in close relation to cirrhosis severity. Limited reports have observed a relationship between platelet count and different etiologies of cirrhosis. Clinically relevant thrombocytopenia in advanced liver fibrosis stages or cirrhosis may result from etiologies other than hypersplenism secondary to portal hypertension alone. These etiologies may additionally include bone marrow suppression attributable to either the virus itself or interferon treatment, alcoholism, dysregulated immune system with the presence of antiplatelet antibodies or immune complexes directed at platelets while potentiating premature clearance of platelets, hepatic sinusoidal dysfunction, alcoholic platelet toxicity, and TPO deficiency due to impaired liver reserves. As was mentioned earlier, splenic destruction of platelets occurs rather than mere splenic pooling; this is supported by the fact that partial splenic embolization can increase platelet counts. Kinetic studies on radiolabeled platelets have provided evidence of splenic pooling and shorter platelet survival. As clinically significant portal hypertension and hypersplenism can persist after antiviral treatments in many patients with baseline cirrhosis, thrombocytopenia may persist in such patients after viral eradication. Medications, including interferon, used to treat CHB or CHC can also impair thrombopoiesis. Ribavirin can induce hemolytic anemia with concurrent, reactive thrombocytosis in patients with CHC [57]. Erythropoietin is an in vivo residual thrombopoietic factor in the absence of the TPO/Mpl pathway. Erythropoietin and TPO together regulate platelet size, both in physiology and under stress. In addition, DIC or consumption coagulopathy is another clinical feature necessitating exclusion in patients with CLD. DIC, not a primary disease, is a thrombohemorrhagic syndrome or complication of various disease entities triggered by infectious and noninfectious pathologies. These etiologies can cause pathologic activation of the extrinsic or intrinsic coagulation pathways and thrombocytopenia. Triggering of blood coagulation follows intravascular expression of tissue factor or activation of the contact pathway in response to pathogen-associated or host-derived, DAMPs. This process is further amplified through inflammatory and immunothrombotic pathways. Activated monocytes and neutrophils are two major inducers of immunothrombosis when they detect PAMPs and DAMPs. Detection of PAMPs and DAMPs triggers tissue factor expression on monocytes and neutrophil extracellular traps released by neutrophils, while promoting immunothrombosis. The consumption of anticoagulants dysregulates homeostasis and thereby disseminates microvascular thrombosis. A tremendous list of clinicopathologic features for triggering DIC must be considered while implementing the platelet count-based monitoring of liver diseases. These mechanisms include the release of tissue factor or thromboplastic substances into the circulation (activation of extrinsic pathway of coagulation), injury to endothelial cells exposing subendothelial collagen (activation of intrinsic and extrinsic pathways of coagulation), systemic hypercoagulopathy (renal disease with loss of antithrombin III), and hepatic disease (decreased synthesis of coagulation factors and anticoagulants). Secretion of tissue factors or thromboplastic substances is often activated by sepsis, severe tissue destruction (trauma, burns), pancreatitis, neoplasms (mucinous carcinomas, leukemia, lymphoma, hemangiosarcoma), and obstetric complications. Endothelial injury can initiate DIC by releasing tissue factor and promoting platelet aggregation to exposed collagen. The causes of endothelial injury may include vasculitis due to the deposition of antigen–antibody complexes (lupus erythematosus and feline infectious peritonitis), temperature extremes (heat stroke and burns), and direct damage by trauma, toxins, rickettsiae, bacteria or their toxins, and viruses [32]. One more underlying feature to exclude (or include in the decision algorithms) is HIT in relation to the mandatory use of unfractionated and low-molecular-weight heparin in clinical practice, including anticoagulation for extracorporeal membrane oxygenator circuits, and dialysis. High-quality evidence about HIT from randomized controlled trials is sparse. In the well-known context of the onset and progression of chronic liver failure, patients with dysregulated balance in coagulation are at risk of thrombophilic, rather than hemorrhagic, conditions. Anticoagulant uses may also be encountered for the recanalization of portal vein thrombosis. Continued heparin use places patients at rapid-onset or delayed-onset risks of developing a potentially devastating immune-mediated thrombophilic disorder caused by induced antibodies against complexes of PF4 and heparin (anti-PF4/H antibodies). An intense hypercoagulable state is triggered by cellular activation of anti-PF4/H antibodies principally engaging the cellular Fcγ receptor, FcγRIIA, on platelets, monocytes, and neutrophils or indirect activation of the endothelial cells through non-FcγR mechanisms. The binding of HIT ultra-large immune complexes to platelet FcγRIIA initiates the phosphorylation of ITAMs and downstream signaling via spleen tyrosine kinase, leading to the degranulation of alpha and dense granules with release of additional PF4, polyphosphate, and ADP and release of soluble P-selectin and microparticles. The released ADP binds to the G-coupled receptor P2Y12 and triggers further activation signals of platelets downstream of Gα_i_ family-dependent intracellular signaling. Negative regulations include the increased expression of TULA-2 and polymorphisms of the tyrosine phosphatase CD148. TULA-2 inhibits the platelet FcγRIIA signaling pathway and HIT in mice. CD148 polymorphisms affect platelet activation and probably exert a protective effect on the risk of HIT in patients with anti-PF4/H antibodies. Systematic results support clinical recommendations regarding platelet count monitoring for HIT [58]. Although PF4-dependent enzyme-immunoassays are sensitive for diagnosing HIT, these assay results, usually not available for hours or days, frequently detect nonpathogenic antibodies in heparin-exposed patients (i.e., low specificity). Accordingly, HIT autoantibody detection with low specificity or positive predictive value should not be routinely performed and should be carefully interpreted in intermediate–low-probability clinical situations [59]. A study employing a US Food and Drug Administration-cleared rapid, automated IgG-specific chemiluminescence-based immunoassay to test stored blood samples demonstrated a high sensitivity/specificity tradeoff for a PF4-dependent immunoassay. This assay assists in platelet-assisted CLD monitoring due to its rapid, on-demand test capabilities, with results being available within 30 min [60].

Longitudinal changes in absolute platelet counts typically have an inverse relationship with those in liver stiffness values on and off (antiviral) treatments in CHB and CHC. However, the impact of posttreatment factors not specific to the regressed liver pathology alone on platelet counting is frequently sustained after treatment. Kinetics (typically increases over time) in platelet counts or platelet count-based diagnostic indices (typically decreases in FIB-4 index and aspartate aminotransferase to platelet ratio index) are not as accurate as liver stiffness measurements beyond the time point of virologic eradication to reflect concurrent liver disease severity. Routine use of noninvasive tests, including platelet-based diagnostic indices after viral eradication in patients with CHC with cirrhosis, has a high false negative rate. Therefore, platelet counts cannot be routinely used to determine which patients no longer need HCC screening or for the diagnosis of cirrhosis reversal [5]. Moreover, platelet count suffers from missing measurements and data in routine clinical settings because follow-ups on platelet count are not currently guideline-regulated. Platelet count and liver stiffness values do not share completely identical underlying, explanatory correlates; therefore, kinetics of both markers over time frequently exhibit distinct discrepancies in cohorts with CHB or CHC both on- and off-treatment. Residual or persistent posttreatment hypersplenism with an unpredictable intrasplenic reservoir size of platelets and clinically significant extrahepatic, posttreatment portal hypertension may cause the utility of the in vitro platelet count to overrate the concurrent CLD severity. A combination of tests may enhance diagnostic or predictive performance. Notably, the utility of platelet count in CLD monitoring may also require cautious interpretation in patients with subclinical and proven HCC. Thrombocytosis is associated with more aggressive tumor biology in many malignancies. Thrombocytosis is a rare paraneoplastic condition seen in HCC. Thrombocytosis in HCC is associated with a high tumor burden, portal vein thrombosis, serum AFP levels, and poor prognosis. Multivariable Cox regression analyses in a cohort study on large-scale patients with newly diagnosed HCC, thrombocytosis (adjusted hazard ratio, aHR = 1.40; 95% confidence interval, CI = 1.23–1.60), and thrombocytopenia (aHR = 1.13, 95% CI = 1.04–1.23) were significantly associated with worse patient survival [61].

### 3.3. Immature Platelet Fraction Measurement

Immature or young platelets are newly released platelets from the bone marrow. Young platelets typically contain large amounts of vestigial remnants of RNA, which can be used in protein synthesis of platelets and can be detected using fluorescent dyes. Young platelets are larger and more reactive than mature platelets and are retained in the circulation for only 24–36 h, during which time a progressive degradation of platelet RNA and a decrease in platelet volume occur [41]. The counting of young platelets provides a fundamental and practical evaluation of the more reactive platelets in anticoagulated peripheral venous blood to indicate concurrent thrombopoietic activity. In a similar manner to reticulocytes in circulation, which are a valid marker of erythropoiesis in bone marrow, young platelets are a promising physiological marker of bone marrow thrombopoiesis after birth (Table 1). The two types of fully automated hematology analyzers include fluorescence-based measurement of young platelets. The terms immature platelet fraction (IPF) and reticulated platelets are not completely synonymous in measurements. The noninvasive patient parameters of “IPF” (IPF%; measured on, e.g., Sysmex XE-/XN-series, Sysmex Corporation, Kobe, Japan) as well as “reticulated platelets” (retPLT%; measured on Abbott CELL-DYN Sapphire/Alinity HQ, Abbott Laboratories, Abbott Park, Illinois, USA) have had a high clinical utility in the differential diagnosis of types of thrombocytopenia [38]. The proprietary RNA fluorescence dye is oxazine-based for Sysmex analyzers and CD4K530 for Abbott analyzers. Sysmex’s XN-Series hematology analyzers offer two diagnostic parameters: absolute immature platelet count (IPF#) and IPF%. IPF% was calculated using the following formula: (particle count in the IPF zone/the particle count in the platelet zone) × 100. The potential limitations (Table 2) of these specialized analyzers include the current lack of a standardized reference method, lack of correlation between the analyzers, and the existence of different reference ranges for different analyzers. IPF is also correlated with platelet size and is an alternative to impedance-derived mean platelet volume (MPV) for diagnosing hereditary thrombocytopenia with large platelets. The highest values of IPF in these hereditary diseases are not related to an increase in intraplatelet RNA content but to the staining of mitochondria and large granules. IPF measurements can be affected by contamination from other blood cells, including leukocyte fragments containing larger quantities of RNA, or by the nonspecific binding of dyes to erythrocyte fragments. A new dye that mainly stains mitochondria and cytosolic RNA and not the plasma membranes of platelets and erythrocytes can preclude staining interferences [41]. Moreover, a falsely elevated IPF% in cold-stored samples carries the risk of potential deviation in interpreting the current thrombopoietic activity. The effect of 4 °C storage was reported to be linear over a 24 h period and independent of the initial IPF% [42]. An algorithm was proposed for a corrected IPF%, which is equal to the uncorrected IPF% minus 1.34 times the length of time in hours of cold storage at 4 °C. However, extrapolating the algorithm is essential for its application to patient cohorts worldwide with long-term cold-stored platelets alone.

IPF can be used to differentiate between the etiologies of thrombocytopenia [42]. IPF increases in disorders that exhibit increased platelet destruction or consumption, and IPF decreases in bone marrow failure with poor intrinsic thrombopoietic activity. IPF has been used as a sensitive indicator of platelet recovery when monitoring patients after chemotherapy and stem cell transplantation. IPF may also serve as a significant feature in decision-making for platelet transfusions, a predictor of bleeding and responses to thrombopoietic agents in patients with peripheral immune-mediated thrombocytopenia. Notably, the absolute IPF count is associated with prognosis in coronary artery disease and stroke as well as resistance to antiplatelet therapy. The immature, more reactive platelets have high prothrombotic potential and are more resistant to functional inhibition by aspirin and P2Y12 receptor antagonists. Young platelets also play a role in the early diagnosis of infectious diseases and the prediction of sepsis [43]. Elevated initial and peak values of percentage IPF and absolute immature platelet counts served as prognostic biomarkers associated with progression to severe conditions in 678 patients with well-characterized COVID-19 [44].

The IPF% was inversely correlated with concurrent peripheral platelet counts in a retrospective cross-sectional study of 105 patients with CHC with detectable serum hepatitis C virus RNA [40]. The absolute IPF count is a real-time gauge of residual platelet reactivity. However, validation in larger prospective trials is necessary to establish the clinical benefits of applying IPF to liver disease monitoring. In a preliminary study [45] including 88 hospitalized patients with cirrhosis of unspecified etiology, the mean values of IPF% (measured on a Sysmex XE-5000 hematology analyzer) varied significantly (*p* = 0.044) among cirrhosis substages: Child-Pugh classes A–C. The IPF% higher than the cutoff value of 3.85% had a sensitivity of 76.6% and a specificity of 52.4%, with an area under the under receiver operating curve characteristics curve of 0.669, for the presence of endoscopically diagnosed esophageal varices.

Another preliminary cross-sectional study included 80 patients with cirrhosis of unspecified etiology and evaluated the performance of noninvasive platelet-assisted diagnosis of esophageal varices. Similarly, IPF% by the Sysmex XE-5000 hematology analyzer was inversely correlated with platelet counts (*p* < 0.001) and positively correlated to Child–Pugh scores (*p* < 0.001). The IPF% was significantly higher (*p* < 0.001) in patients with cirrhosis with treatment-naïve esophageal varices diagnosed through endoscopy than in patients with cirrhosis without varices. IPF% could detect the presence of esophageal varices at a cutoff level of 12% in patients with cirrhosis. The sensitivity was 97.5%, specificity 97.5%, positive predictive value 97.5%, negative predictive value 97.5%, and accuracy 99.3% for the detection of varices [46]. However, the utility of the useful marker IPF in the monitoring and prognosis of CLD from pretreatment and posttreatment baselines to the surveillance period beyond the treatment period is warranted in prospective CLD cohorts worldwide.

### 3.4. TPO Level Quantitation

TPO is produced in the liver, kidneys, and bone marrow at a constant physiological rate. TPO can synergize with other cytokines, such as IL-3, IL-6, IL-11, erythropoietin, and granulocyte colony-stimulating factor, to promote the development and differentiation of megakaryocytes and erythroid lineage [62]. TPO may also potentiate platelet activation to compensate for thrombocytopenia [4]. However, upon the binding of TPO to TPO-R on megakaryocytes, thrombocytes, and stem and progenitor cells, TPO is internalized and cleared from the circulation (Table 1). The net effects on blood TPO levels also come from the clearance of TPO from the circulation by the size of the entire platelet reservoir, including immeasurable intrasplenic platelets. In CLDs, the physiological or pathological inverse correlation between blood TPO and platelet levels is not applicable. Therefore, blood TPO levels cannot be employed as clinically relevant predictive markers in CLD monitoring (Table 2) [40].

### 3.5. MPV Measurement

The normal MPV range is approximately 9–12 femtoliters in adults. There is laboratory-to-laboratory discordance in normal ranges of the routinely reported values of MPV in clinical blood tests due to discrepancies in the hematology analyzers, techniques, and anticoagulants used. Several studies have reported a significant correlation between MPV and nonalcoholic fatty liver disease, while others have not. The discrepancies in MPV results measured using hematology analyzers could be partially attributed to the different measurement methods used by the analyzers, including impedance and light diffraction [38]. Technical incapacities (Table 1), including anticoagulant selection (citrate or EDTA), anticoagulant-induced increase in platelet volume in relation to altered platelet morphology over time [47], and the absence of the standardization of MPV among different technologies have also hindered the utility of MPV as a valid marker in platelet-assisted CLD monitoring to date (Table 2) [48].

### 3.6. Platelet Function Testing

#### 3.6.1. Preanalytical, Technical, and Analytical Variables

Platelet aggregometry is designed to evaluate concurrent, in vitro platelet function in terms of primary hemostasis capacity and guide antiplatelet therapy and surgery. By measuring the increased optical-based light transmission, the historic, widely used gold standard aggregometry (LTA) evaluates the in vitro aggregation of peripheral blood platelets [63]. Platelet aggregation can occur in positive response to different available platelet agonists, commonly including ADP, collagen, and epinephrine, at different concentrations for individual pathway dissection. However, LTA requires the processing of large volumes of citrated platelet samples in suspension and strict expertise and interpretation. LTA is also sensitive to platelet counts and antiplatelet therapies. Multiple-electrode aggregometry is a variation of traditional platelet aggregometry that measures changes in electrical impedance. Similar to LTA, impedance aggregometry can also be used to measure platelet function in whole blood. Upon activation by agonists, the increase in platelet aggregation to the sensor wires is proportional to the increase in electrical resistance. However, platelet counts and hematocrit can affect the measurement results. In vitro whole-blood aggregometry mimics the in vivo conditions of platelet activation and aggregation. Whole-blood aggregometry relies on platelet counts and therefore does not allow for the comparison of thrombocytopenic patients with cirrhosis with healthy participants with normal platelet counts. Study results on platelet aggregation in cirrhosis have yielded conflicting results because the interpretation of platelet functional testing was challenged by thrombocytopenia. Combined with luminescence, platelet lumiaggregometry, a less commonly used semiquantitative assay, is useful in the combined valuation of platelet granule release and storage disorders. The VerifyNow system is a simple yet expensive, fully automated point-of-care test equipped with disposable cartridges containing platelet agonists and fibrinogen-coated beads. PFA (PFA-100 and PFA-200, Siemens Healthcare Diagnostics, New York, NY, USA), a simple, automated, and rapid whole-blood test, requires minimal volume of blood but is also affected by platelet counts and hematocrit. PFA measures the closure time of a membrane aperture through adhesion and aggregation of the platelets under flow conditions mimicking a high shear flow through the blood vessels. It provides point-of-care screening for platelet function disorders and monitoring antiplatelet therapies. It has a high negative predictive value and may be used as a screening test for von Willebrand’s disease and platelet dysfunction. Prolonged membrane closure time may require specific testing for these disorders. The PFA-100 is the most used primary hemostasis-screening instrument and has been remodeled and upgraded to the PFA-200.

Platelet preparation for basic or clinical studies employs whole blood, platelet-rich plasma, and washed or gel-filtered platelets. Platelets are also highly sensitive to preanalytical spurious platelet activation by changes in temperature, pH, shear, or mechanical stress, artificial materials and chemicals, and additive reagents after removal from their natural microenvironment in the circulation (Table 1). Therefore, blood should be judiciously drawn into polypropylene plastic or silicone-coated tubes with the recommended sodium citrate buffer. The anticoagulant recommended for washed platelets is an acid–citrate–dextrose buffer (pH 6.5). While chelating calcium ions, a lower pH prevents platelets from activation during isolation. Moreover, the inhibitory prostaglandin I2, prostaglandin E1, and apyrase can be added to platelet-rich plasma before centrifugation to prevent platelets from activation or aggregation during centrifugation or the washing process. Clinical features such as exercise, smoking, caffeine, and medications should also be considered before reporting the finalized aggregometry results. Additional challenging features to incorporate into a decision algorithm include the need for high blood volumes and the interference of low platelet counts and hematocrit. Platelet counts below 30 × 10^9^/L in CLD pose challenges to testing [21].

Liver dysfunction biomarkers may reflect an increasing prothrombotic profile in patients with cirrhosis, exhibiting a relative decrease in endogenous anticoagulants compared with procoagulants in previous studies [49]. Moreover, hyperaggregability or hypoaggregability can be rigorously rebalanced during cirrhosis [64]. Defective in vivo platelet function, commonly reflected by the debated aggregability, arises from the activation of inhibitory pathways intrinsic to the platelets and through interactions of platelets with responsible circulating cells and plasma agonists and antagonists. Underlying mechanisms dependent on types of liver diseases and kinetics in liver fibrosis stages and the liver microenvironment may exert opposing effects. The net effects have resulted in contradictory results in in vitro platelet aggregability studies, which currently lack investigation standards. In cirrhosis, increased platelet aggregation may counterbalance low platelet counts with concurrently high circulating vWF levels. This may explain the nonsignificant correlation between thrombocytopenia and bleeding in cirrhosis exhibiting rebalanced hemostasis. International guidelines are therefore against the routine correction of thrombocytopenia in patients with cirrhosis undergoing invasive procedures. Conventional platelet functional assays are not sensitive to platelet phenotypic heterogeneity and interactions with circulating cells or factors in parallel with kinetics of concurrent liver disease severity. Conventional aggregability testing investigates aggregation in static conditions. Additional platelet functions, including adhesion and secretion, also independent of thrombocytopenia, require further investigation. Aggregability testing is thus insufficient to fully delineate the interplay between multimers of vWF, platelets, and blood vessel walls under in vivo high-flow conditions. To enhance platelet function monitoring in validity and reliability in platelet-assisted CLD monitoring, advances in the utility of specialized analyzers, electron microscopy, flow cytometry, mass spectrometry and ‘omics’ studies, and various molecular biological techniques are therefore warranted [50].

#### 3.6.2. Applications in CLDs

Studies on platelet aggregation employing whole blood in cirrhosis are controversial because the interpretation of platelet dysfunction is challenged by thrombocytopenia in relation to cirrhosis (Table 2). However, there has been increasing demand for CLD monitoring through platelet function testing. The dysregulated upregulation in platelet aggregability in patients with decompensated cirrhosis may predispose these patients to the risk of either prothrombotic adverse events, including acute myocardial infarction, or risk of bleeding events. The administration of TPO receptor agonists and transfusion of platelets may also complicate the already increased platelet activation, aggregation, and ultimate thrombosis, especially in decompensated cirrhosis. The results of a recent study [17] are critical to the application of platelet-assisted CLD monitoring. The utility of baseline platelet aggregation normalized to platelet count was thoroughly applied to unmatched case–control comparisons of concurrent CLD severity and cirrhosis substages and to time-dependent risk stratification for liver-related adverse events over time. In a prospective study on 203 patients with cirrhosis (decompensated n = 157; 77%), a ratio between platelet aggregation assessed using aggregometry (Multiplate) of peripheral venous blood and platelet count (i.e., PLT ratio = [platelet aggregation expressed as area under the curve/platelet count × 10^3^/mL] × 1000) was applied to stratify the enrolled patients in terms of liver disease severity and prognosis. The PLT ratio was significantly higher in the group with cirrhosis (n = 203) than in the control group comprising CHB or CHC (n = 60) and healthy participants (n = 45) (0.44 [interquartile range: 0.26–0.67] vs. 0.25 [0.19–0.31] and 0.26 [0.22–0.31], respectively; *p* < 0.0001). Platelet counts were lower in patients with cirrhosis (90 × 10^9^/L) than in controls with chronic hepatitis (215 × 10^9^/L) and healthy participants (213 × 10^9^/L) (*p* < 0.0001). Similarly, the prevalence and severity of thrombocytopenia were significantly higher in patients with cirrhosis than in controls. Platelet aggregation induced using ADP 6.5 μmol/L (ADP test, Roche Diagnostics GmbH, Mannheim, Germany) was positively correlated with platelet counts in both patients with cirrhosis and controls. The PLT ratio increased significantly with the severity of cirrhosis (Child-Pugh scores for CLDs and cirrhosis: scores C > B > A in PLT ratios). The PLT ratio was significantly higher in decompensated patients with severe thrombocytopenia. In decompensated patients, the PLT ratio is associated with cirrhosis progression. Among decompensated patients (n = 157), 65 (41%) developed liver-related adverse events, including further decompensation, need for liver transplantation, and mortality during a median follow-up of 118 days. A multivariate Cox regression analysis identified PLT ratio (aHR = 1.87, 95% CI = 1.23–2.84; *p* = 0.003) and model for end-stage liver disease score (aHR = 1.05, 95% CI = 1.01–1.08; *p* = 0.01) as significant predictors for the development of defined liver-related adverse events. The relative risk of events was 7.5-fold higher in patients with a PLT ratio >0.75 versus patients with a PLT ratio < 0.25 (relative risk: 7.5, 95% CI: 2.5–21.9; *p* = 0.003). However, unrecognized subclinical [65] and rapid changes and recurrence in hepatic decompensations in patients with cirrhosis is a kinetic course that may change rapidly. Longitudinal platelet function evaluations are required to provide a more thorough assessment over time. In future studies, the reference test should be a continuous variable rather than ordinal scoring, such as Child-Pugh scores. Moreover, LTA, the gold standard for measuring platelet function, is recommended for platelet counts above 150 × 10^9^/L. A specialized flow cytometric approach [51] is thereby highly warranted to quantify real-time platelet function to judiciously monitor the unstable counterbalancing between hyperaggregable and hypoaggregable as well as hypercoagulable and hypocoagulable states in thrombocytopenic settings in CLDs at risk of prothrombotic or bleeding adverse events over time. However, platelet aggregation was potentiated by cold storage and diminished by cryopreservation (at −80 °C). Cryopreserved conditions significantly decrease platelet activation and severely abrogate platelet aggregation while exhibiting a procoagulant phenotype compared with platelets stored at room temperature [47]. Therefore, the use of archived blood samples is hindered to date in retrospective studies on cohorts worldwide. The development of a correction algorithm is warranted. Larger-scale prospective longitudinal studies including patients with CLD are required to validate the results on various platelet function tests while stratifying patients by the risk of LREs from baselines before and after (antiviral) treatments.

## 4. Conclusions

Compared to the performance of liver stiffness measurement, the impact of extrahepatic factors on platelet count-based diagnostic approaches after CLD treatment needs to be incorporated into the decision algorithms and prediction models in CLD monitoring. IPF is more reliable and valid than MPV. A point-of-care approach is essential to quantify the real-time platelet function to judiciously monitor the unstable counterbalancing between hyperaggregable and hypoaggregable states in CLDs at risk of prothrombotic or bleeding adverse events over time. This approach assists in stratifying patients according to their risk of LREs from baselines before and after CLD treatment.

## Figures and Tables

**Table 1:** Physiological and pathological mechanisms, clinical utility, and clinical and analytical interferences in the context of platelet-assisted CLD monitoring.

Measurement	Mechanism	Clinical Application	Interferences	
Liver stiffness	Intrahepatic extracellular matrix’s collagen content Dynamic component of hepatic sinusoidal pressure consisting of blood flow, intrahepatic resistance, and hemorheology Static component of hepatic sinusoidal pressure related to the vasculature elasticity and intravascular volume filling status Intrahepatic intracellular pressure through transport proteins and aquaporins Stretch forces on the cellular membranes and intermediary filaments of the parenchymal hepatocytes and nonparenchymal hepatic stellate cells and liver sinusoidal endothelial cells	Concurrent pretreatment (viral) liver fibrosis staging Concurrent posttreatment liver fibrosis staging Cirrhosis substaging HCC prognosis LRE prognosis from pretreatment and posttreatment baselines	Sensitivity to spurious acoustic attenuation Augmentation by concurrent hepatic necroinflammation, congestion, cholestasis, alcohol consumption, postprandial state Ascites Body mass index Valsalva maneuver	[34,35,36,37]
Platelet count	TPO ligand targeting c-Mpl proto-oncogene-encoded protein receptor, TPO receptor on megakaryocytes Bone marrow progenitor cells and niche Acute-phase reaction In vivo binding of Ashwell–Morell receptor of hepatocytes with desialylated platelets to regulate hepatic TPO production through activation of JAK2/STAT3 Platelet sequestration Immune and nonimmune platelet clearance Binding of hepatic Ashwell–Morell receptor or other scavenger receptors on hepatocytes and macrophages with desialylated platelets to remove desialylated platelets Balance between the proapoptotic and antiapoptotic signaling pathways in platelets Overactivation of platelets	Liver fibrosis staging Cirrhosis substaging HCC prognosis LRE prognosis Variceal exclusion	*Preanalytical:* Anticoagulants Platelet satellitism Platelet clumping Cold storage *Technical:* Extreme platelet sizes Blood cell fragments, cryoglobulins, lipids, and pathogens Anticoagulants *Clinical:* Increasing counts: thrombocytogenic medications, anemia with increased erythropoietin activity, systemic infections, inflammation, and underlying cancer Decreasing counts: posttreatment residual hypersplenism, clinically significant extrahepatic portal hypertension, bone marrow infection, suppression, alcohol toxicity, disseminated intravascular coagulation, and heparin-induced thrombocytopenia	[5,6,38,39]
IPF	Immature platelets containing vestigial RNA capable of binding fluorescent dyes Young and more reactive platelets in peripheral blood reflecting physiological and pathological bone marrow thrombopoietic activity Increased prothrombotic potential Resistance to functional inhibition by aspirin and P2Y12 receptor antagonists	Thrombocytopenia discrimination Cirrhosis substaging Variceal detection	Cold storage Storage duration Platelet size Contamination of fluorescent staining of fragments containing large quantities of RNA from leukocytes and erythrocytes Requirement on new dye mainly staining mitochondria and cytosolic RNA and not plasma membrane of platelets and erythrocytes Discrepancy in measurement results between analyzers Different reference ranges for analyzers	[40,41,42,43,44,45,46]
Circulating TPO level	A glycoprotein cytokine ligand targeting c-Mpl proto-oncogene-encoded protein receptor, TPO receptor Constant production in the liver, bone marrow, and kidneys by both parenchymal cells and liver sinusoidal endothelial cells In vivo binding of Ashwell–Morell receptor of hepatocytes with desialylated platelets to regulate hepatic TPO production Internalization and clearance from the circulation upon the binding of TPO-to-TPO receptor on megakaryocytes, thrombocytes, stem cells, and progenitor cells	Thrombocytopenia discrimination Treatment response prediction for TPO receptor agonists	Diurnal variation in blood levels Increase in thrombocytopenia Clearance by platelet reservoir size, including immeasurable intrasplenic platelets Decreased production in advanced CLDs	[2]
MPV	Average platelet size Connection to productive and consumptive states of platelets and platelet aggregation	Thrombocytopenia discrimination Platelet aggregation evaluation	Anticoagulant selection, anticoagulant-induced increase in platelet volume over time, changes in platelet morphology, absence of test standardization Chronic myeloid leukemia, hyperthyroidism Immune thrombocytopenic purpura, preeclampsia Renal failure Aplastic anemia, chemotherapy Infection, inflammation, cancer	[38,47,48]
Platelet aggregation	Nitric oxide and prostaglandin I2, released by healthy vascular endothelium, increasing the levels of cGMP and cAMP in platelets, and inhibiting platelet activity by activating protein kinase A and protein kinase G Intertwined cross talks between inhibitory and activatory cascades A positive response to activators including adhesive proteins, soluble agonists, and environmental factors Modes of signal transduction downstream of their respective platelet receptors Divergent early receptor signaling pathways converged through common downstream amplifying signaling pathways like a hub Inside-out signaling mode with binding of cytosolic adapter protein talin and kindlins to αIIbβ3 causing conformational change of extracellular domain of αIIbβ3 to exhibit a high affinity for activators Integrin outside-in signaling by talin reassociation with β3 cytoplasmic domain and calpain cleavage of Src binding site in the β3 tail	Counterbalance evaluation between hyperaggregable and hypoaggregable states in CLDs Risk estimation of prothrombotic or bleeding events in CLDs Cirrhosis substaging	Microenvironmental factors Temperature, pH, shear, mechanical stress, artificial materials, chemicals, additive reagents Platelet count Cold storage Cryopreservation Patient age Smoking	[13,18,19,20,21,40,49,50,51]
P-selectins	Single-chain transmembrane glycoproteins A family of cell adhesion molecules (CD62P) located in the α-granules of platelets and Weibel-Palade bodies of endothelial cells Retention in the platelet granules, mobilized to platelet surface when activated by platelet agonists A special index of platelet activation	Liver reserve evaluation	Clones for CD62P and IgG monoclonal antibody Conformity between CD62P staining and fixing protocols and subsequent analysis Conformity between flow cytometers	[52,53]

CLD, chronic liver disease; HCC, hepatocellular carcinoma; IPF, immature platelet fraction; LRE, liver-related event; MPV, mean platelet volume; TPO, thrombopoietin.

**Table 2:** Advantages and disadvantages, pitfalls, or limitations of marker utility in patients with chronic liver diseases.

Liver Stiffness
*Advantages* As a continuous variable, it closely indicates the real-time combo gauge of liver status in synchrony with concurrent intrahepatic extracellular matrix, sinusoidal pressure, and intracellular pressure Helps with concurrent, promising, and validated liver fibrosis staging Serves as a promising predictor for LREs and HCC from pretreatment (viral) and posttreatment baselines
*Disadvantages* Sensitive to measurement interferences Confounded by hepatic features including pretreatment and concurrent hepatic necroinflammation
**Platelet Count**
*Advantages* As a continuous variable, serves as a valid indirect marker of liver fibrosis Helps with promising, pretreatment, and concurrent liver fibrosis staging Helps exclude the presence of concurrent high-risk varices to reduce unnecessary variceal screening endoscopies Serves as a promising predictor for the incidence of LREs and HCC over time from a pretreatment baseline
*Disadvantages* May exhibit distinct kinetics not in parallel with those of liver stiffness on and off (antiviral) treatment May be affected by kinetics in the on-treatment and off-treatment effects of extrahepatic pathogenesis confounding the utility of platelet count in CLD monitoring May overrate the concurrent CLD severity with excessive thrombocytopenia due to residual or persistent posttreatment hypersplenism with an unpredictable intrasplenic reservoir size of platelets May overrate the concurrent CLD severity in case of residual or persistent posttreatment clinically significant, extrahepatic portal hypertension Complicated by spurious reporting, including pseudothrombocytopenia Not sufficiently sensitive or valid to implement posttreatment predictions
**Immature Platelet Fraction**
*Advantages* Clinically available Avoids unnecessary bone marrow biopsy Assists with evaluating the real-time, residual platelet reactivity Differentiates between cirrhosis substages Differentiates reliably in thrombocytopenic settings
*Disadvantages* Lacks a standardized reference method Exhibits potential discrepancy of measurement results between analyzers Interfered with by different reference ranges for different analyzers Poses the risk of potential deviation in the interpretation of the current thrombopoietic activity by a falsely elevated value reported in cold-stored samples Pending a correction algorithm for values reported in long-term cold-stored samples Pending monitoring and prognosis of CLDs in prospective CLD cohorts worldwide Complicates the use of long-term cryopreserved blood samples in a retrospective study on cohorts worldwide
**Thrombopoietin Level**
*Advantages* Capable of being quantified through newly developed fully automated quantitative assays combining high sensitivity and high throughput Helps distinguish between causes of thrombocytopenia and predict treatment response to thrombopoietin receptor agonists Helps identify advanced CLD with decreased thrombopoietin levels
*Disadvantages* Not feasible in routine clinical measurements Possesses net effects from the compensatory (inversely correlated) increase in thrombocytopenia, clearance by platelet reservoir size including immeasurable intrasplenic platelets, and decreased production in advanced CLDs Lacks the physiological or pathological predictable inverse correlation between blood thrombopoietin levels and platelet counts in CLDs Cannot be employed as one of the predictive markers to date to exhibit an adequate discriminatory capacity in CLD monitoring
**Mean Platelet Volume**
*Advantages* Differentiates between hematological disorders, along with utility of concurrent platelet count
*Disadvantages* Pending solutions to technical incapacities including anticoagulant selection, anticoagulant-induced increase in platelet volume in relation to changes in platelet morphology over time, and the absence of standardization of measurement among different technologies Inferior to immature platelet fraction in discrimination between the causes of thrombocytopenia because younger platelets are not necessarily larger Cannot be employed as one of the predictive indicators to date to exhibit an adequate discriminatory capacity in CLD monitoring
**Platelet Aggregation**
*Advantages* Necessarily monitors the unstable counterbalancing between hyperaggregable and hypoaggregable as well as hypercoagulable and hypocoagulable states in thrombocytopenic settings in CLDs at risk of prothrombotic or bleeding events Differentiates between cirrhosis substages through correction by platelet count
*Disadvantages* Requires a specialized, time-consuming preanalytical and analytical process with well-controlled relevant variables Cannot fully delineate interplays among multimers of von Willebrand factor, platelets, and blood vessel walls under in vivo high-flow conditions through in vitro tests Complicated by cryopreserved conditions, which significantly decreases platelet activation and severely abrogates platelet aggregation Pending a correction algorithm for values reported in long-term cold-stored samples Complicates the use of long-term cryopreserved blood samples in a retrospective study on cohorts worldwide Pending prospective longitudinal studies on patients with CLD of a larger scale for development and validation
**P-Selectin Expression of Platelets**
*Advantages* Correlated with worse liver reserves in limited previous studies requiring updates
*Disadvantages* Pending uniform protocols of flow cytometric analysis Cannot be employed as one of the applicable predictive indicators to date to exhibit an adequate discriminatory capacity in CLD monitoring

CLD, chronic liver disease.

## Data Availability

Not applicable.

## Abbreviations

aHR, adjusted hazard ratio; AMR, Ashwell-Morell asialoglycoprotein receptor; Bcl2, B-cell lymphoma 2; CCl4, carbon tetrachloride; CHB, chronic hepatitis B; CHC, chronic hepatitis C; CI, confidence interval; CLD, chronic liver disease; COVID-19, Coronavirus disease 2019; DAMP, damage-associated molecular pattern; DIC, disseminated intravascular coagulation; EDTA, ethylenediaminetetraacetic acid; HCC, hepatocellular carcinoma; GP, glycoprotein; IL, interleukin; IPF, immature platelet fraction; JAK, Janus kinase; LRE, liver-related event; LSEC, liver sinusoidal endothelial cell; MAPK, Mitogen-activated protein kinase; MPL, myeloproliferative leukemia; MPV, mean platelet volume; PAMP, pathogen-associated molecular pattern; PF4, platelet factor 4; STAT 3, signal transducer and activator of transcription 3; TPO, thrombopoietin; TPO-R, TPO-receptor; TULA-2, T-cell ubiquitin ligand-2; vWF, von Willebrand factor.
